# High prevalence of antimicrobial residues in broiler meat in Bangladesh, India and Vietnam

**DOI:** 10.1038/s41538-026-00806-7

**Published:** 2026-04-04

**Authors:** Ludovic Pelligand, Kelyn Lee Ghee Seow, Saira Butt, Thuy Thi Hoang, Huong Quynh Luu, Son Thi Thanh Dang, Ngoc Thi Pham, Hoa Thi Thanh Pham, Anne Conan, Akash Vinubhai Golaviya, Haidaruliman Ismail Paleja, Prakash Koringa, Ketankumar Joitaram Panchal, Ankit Tulsidas Hinsu, Md. Abu Shoieb Mohsin, Mahmudul Hasan, Mohammed Abdus Samad, Sri Rajiv Kumar Roy, Pangkaj Kumar Dhar, Md. Helal Uddin, Rashed Mahmud, SKM Azizul Islam, Paritosh Kumar Biswas, Md. Ahasanul Hoque, Yeong Cheng Cheah, Yulan Wang, Damer Blake, Dominique Hurtaud-Pessel, Patricia Lynne Conway, Guillaume Fournié, Fiona Tomley

**Affiliations:** 1 Royal Veterinary College, Hatfield, UK; 2 Singapore Centre for Environmental Life Sciences Engineering (SCELSE), Nanyang Technological University, Singapore, Singapore; 3 Sydney School of Veterinary Sciences, University of Sydney, Sydney, NSW Australia; 4 Disease Ecology Laboratory, Sydney School of Veterinary Sciences, University of Sydney, Sydney, Australia; 5 National Institute of Veterinary Research, Ha Noi, Vietnam; 6 Vietnam Institute of Animal and Veterinary Science (VIAVS), Hanoi, Vietnam; 7 CIRAD (French Agricultural Research Centre for International Development), Ha Noi, Vietnam; 8 Centre for Applied One Health Research and Policy Advice, City University of Hong Kong, Hong Kong SAR, China; 9 CIRAD, Harare, Zimbabwe; 10 ASTRE unit, Univ Montpellier, CIRAD, INRAE, Montpellier, France; 11 Department of Veterinary Biotechnology, College of Veterinary Science and Animal Husbandry, Kamdhenu University, Anand, India; 12 Chattogram Veterinary and Animal Sciences University, Chittagong, Bangladesh; 13 Bangladesh Livestock Research Institute, Savar, Dhaka, Bangladesh; 14 Singapore Phenome Center, Lee Kong Chian School of Medicine, Nanyang Technological University, Singapore, Singapore; 15 Human Phenome Institute, Fudan University, Shanghai, China; 16 ANSES (French Agency for Food, Environmental and Occupational Health & Safety), Laboratoire de Fougères, Fougères, France; 17 School of Biological, Earth and Environmental Sciences (BEES), UNSW Sydney, Sydney, NSW Australia; 18 INRAE, VetAgro Sup, UMR EPIA, Université de Lyon, Marcy l’Etoile, France; 19 INRAE, VetAgro Sup, UMR EPIA, Université Clermont Auvergne, Saint Genès Champanelle, France; 20 Present Address: Vietnam Institute of Animal and Veterinary Science (VIAVS), Hanoi, Vietnam

**Keywords:** Biotechnology, Environmental sciences, Microbiology

## Abstract

We screened 558 chicken breast meat samples from farms (at the end of production) and endpoints (sale/slaughter outlets) in India (Gujarat state), Bangladesh, and northern Vietnam for 69 veterinary antimicrobials and tested outcomes with the EU maximum residue levels. The prevalence of non-compliant samples declined from farms to endpoints, where it was 3.7%-8.6% (vs 0.05–0.09% in the EU for 2021-2022). Strengthened residue testing and stakeholder awareness of withdrawal periods are needed as poultry production intensifies.

## Evidence gap in regions where systematic residue surveillance is scarce

Demand for animal-source food is increasing globally, particularly in South and Southeast Asia^1^. This growth is accompanied by intensification of livestock production, especially chickens, often with increased antimicrobial (AM) use^2^. Misuse of AM in chickens may promote resistance, resulting in failure of later treatment and posing risks to human health through (i) selection and environmental dissemination of resistant bacteria^3^, and (ii) active AM residues in meat that may harm consumers directly or select for resistance in the human gut^4^. The EU Antimicrobial Expert Group (AMEG) classifies veterinary AMs based on the potential risk they pose to human health^5^: AMs used in poultry include those from the lowest risk category, D (penicillins, sulfonamides/trimethoprim, and tetracyclines), up to critically important classes for human health in categories C (aminoglycosides, macrolides) and B (3^rd^/4^th^ generation cephalosporins, fluoroquinolones, colistin).

To ensure consumer safety in the EU, maximum residue levels (MRLs) are defined for each licensed AM. These international standards^6,7^ are based on acceptable daily intakes (ADI) derived from microbiological data relating to the human intestinal flora^4,8^. Use of licensed AMs in chickens is permitted, provided a sufficient withdrawal period (WP) is observed before slaughter to prevent residues reaching beyond limits regarded as safe. Residue surveillance increasingly relies on bioanalytical methods, typically liquid-chromatography mass-spectrometry (LC-MS/MS) validated for the simultaneous detection of multiple AMs at MRLs.

Through a large-scale cross-sectional study in Bangladesh, northern Vietnam, and the Indian state of Gujarat, we assessed (i) the prevalence of non-compliant chicken meat samples (at least one AM > MRL), (ii) variation in prevalence by geographical zone (i.e. study area in each study region), type of premises and production stage, and (iii) agreement between farmer’s self-reported AM use and analytical results.

## Results

**Fig. 1:**
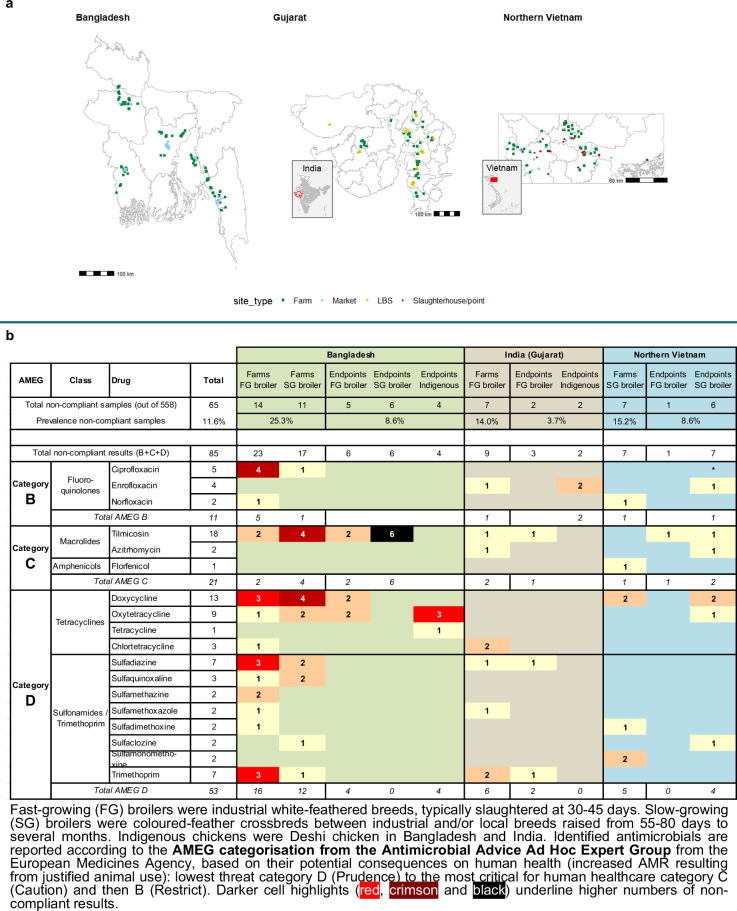
Sampling locations and results from the antimicrobial residue screening assay. a 558 chicken meat samples were analysed from Bangladesh (273 samples from 99 farms and 50 markets), Gujarat (158 samples from 50 farms and 65 live bird shops) and Vietnam (127 samples from 46 farms and 51 endpoints). Site types are represented as green, blue and red dogs for farms, markets and slaughter points respectively. Maps of the locations of sampling sites were produced with GPS coordinates; b non-compliance results summary in 558 chicken meat samples collected from Bangladesh, Gujarat (India), Vietnam. Samples were analysed using the same multidrug analytical technique and thresholds (Maximum Residue Limits MRL) as the EU reference laboratory. Non-compliant samples were samples with ≥1 non-compliant result. A non-compliant result represented detection of at least 1 antimicrobial ≥1x MRL.

We collected 558 chicken meat samples from 362 sites (Figs. 1a, 1b, Supplementary Table 2) in Bangladesh (*n* = 273 samples), Gujarat (India, *n* = 158), and Vietnam (*n* = 127). Chicken types and farm sizes are summarised in Supplementary Tables 3 and 4. Fast-growing broilers were commercial, white-feathered breeds, typically slaughtered at 30–45 days, whereas slow-growing broilers were coloured-feather hybrids of commercial and/or local breeds raised for between 55 days and several months. All samples were screened for 69 AMs and compared with EU MRL concentrations^9^ using a LC-MS/MS platform replicating the EU reference laboratory analytical method^10^ (Supplementary study protocol).

Across all zones, 11.6% of samples were non-compliant (65/558 samples, 95%CI:9.2–14.7), with higher prevalence on farms at end of production (20.0%, 39/195, 95%CI:15.0–26.2) than at endpoints (7.2%, 26/363, 95%CI:4.8–10.4%), with the latter referring to sites where chickens were slaughtered and/or sold to end-users. In Bangladesh, 14.7% of samples (40/273, 95%CI:10.8–19.5%) were non-compliant, including nine with residues exceeding ten times the MRL for at least one AM. In Gujarat, 7.0% (11/158, 95%CI:3.7–12.4%) were non-compliant, including three samples with residues >10×MRL, and in Vietnam, 11.0% (14/127, 95%CI:6.4–18.1%) were non-compliant, including four samples with residues >10×MRL. Prevalence was consistently higher on farms than at endpoints in each zone.

We fitted logistic regression models with sample compliance as the binary outcome to assess variation across strata of the chicken population. Key explanatory variables included zone, site type (farm, endpoint), chicken type (fast-growing broiler, slow-growing broiler, indigenous), and finished status (at or below expected slaughter age/weight). Associations with these variables were assessed both separately and in mutually adjusted models. Time from sampling to testing was controlled for, given potential AM degradation^11^. When considering samples from chickens sampled both on farms and at endpoints, odds of non-compliance were more than three-fold higher in unfinished chickens on farms (OR = 3.4, 95%CI:1.68–6.85) and more than twice as high in finished chickens on farms (OR = 2.3, 95%CI:1.10–4.79) compared with chickens at endpoints (*P* = 0.001) (Table 1, Supplementary Table 5). Although the odds appeared lower for fast-growing broilers in Gujarat (before adjusting for finished status), and for slow-growing broilers in Vietnam compared with fast-growing broilers in Bangladesh (OR = 0.3, 95%CI:0.07-0.98), the joint effect of zone and chicken type was not statistically supported (*P* = 0.133). Models were additionally stratified by site type, with endpoint-specific models including chicken types present at endpoints but absent on study farms. On farms (Supplementary Tables 6), the pattern for zone and chicken type was consistent with the joint farm-endpoint model, while no significant difference was observed between finished and unfinished chickens (*P* = 0.244). At endpoints (Supplementary Tables 7), the difference in the odds of non-compliant samples between Bangladesh and both Gujarat and Vietnam was not significant (*P* = 0.139), and odds did not differ across chicken types, including between indigenous chickens and broilers (*P* = 0.541).

**Table 1:** Logistic regression models were fitted with sample compliance as the binary outcome

	*n*	non-compliant (%)	Univariable OR (95% CI)	*P*	Multivariable OR (95% CI)	*P*
**Farm-endpoint model**						
Zone-chicken type						
Bangladesh - fast-growing broilers	103	18 (17.5%)	1	0.063	1	0.133
Bangladesh - slow-growing broilers	109	18 (16.5%)	0.90 (0.44–1.86)		0.87 (0.41–1.82)	
Gujarat - fast-growing broilers	101	9 (8.9%)	0.39 (0.16–0.92)		0.43 (0.16–1.09)	
Vietnam - slow-growing broilers	111	13 (11.7%)	0.28 (0.07–0.93)		0.28 (0.07–0.98)	
Finished-site						
Finished-market	229	19 (8.3%)	1	0.001	1	0.001
Finished-farm	107	17 (15.9%)	2.08 (1.02–4.19)		2.30 (1.10–4.79)	
Unfinished-farm	88	22 (25.0%)	3.75 (1.91–7.45)		3.37 (1.68–6.85)	

*Univariable* refers to models fitted with one key explanatory variable at a time, and *multivariable* to models fitted with both. Time from sampling to testing was controlled for in all models given potential antimicrobial degradation. Further details on the models fitted to the joint farm–endpoint dataset, as well as to the farm- and endpoint-specific datasets, are provided in the Supplementary Material (Supplementary Tables 4, 5 and 6, respectively).

*OR* Odd Ratio, *CI* Confidence Interval, *P* Likelihood Ratio Test *P*-value.

Among the 65 non-compliant samples, 85 non-compliant results were identified, with 17 samples yielding multiple detections (13 in Bangladesh, three in Gujarat, and one in Vietnam). Most involved sulfonamide +/- trimethoprim (AMEG category D, *n* = 27) and tetracyclines (D, *n* = 26), followed by macrolides (almost exclusively tilmicosin; AMEG C, *n* = 20), florfenicol (AMEG C, *n* = 1), and fluoroquinolones (enrofloxacin, ciprofloxacin, norfloxacin; AMEG B, *n* = 11) (Fig. 1B). However, reporting of levofloxacin use on 13 Bangladeshi farms suggests underestimation of the frequency of fluoroquinolone residues, as levofloxacin was not included in the EU-based LC-MS/MS panel. Treatment on farms, chicken age, and weight for each non-compliant sample are detailed in Supplementary Table 8.

Only 20 of 56 non-compliant results on farms could be explained by farmer-reported ongoing or recent AM use, including 18 in Bangladesh (Supplementary Table 8). Ongoing treatment accounted for only five out of 13 samples with high-concentration (>10×MRL) non-compliant farm samples. For another 6 non-compliant results, administration of the specific AM was recorded but at dates inconsistent with the residue levels (median of 24 days between treatment end date and sampling), suggesting possible unreported re-administration.

## Discussion

Using the harmonised EU reference LC-MS/MS method and MRL thresholds, the proportion of chicken meat samples from endpoints that were non-compliant for AM residues was found to be 50-100-fold higher than in the EU. EU data provide a robust comparator, as member states are legally required to monitor residues of pharmacologically active substances in food-producing animals and food products^12^. During the same time period and for the same AM panel, six EU countries reported 15 non-compliant poultry samples out of 16,877 tested (0.09%)^13^. In the EU, this prevalence has remained below 0.2% and declined over the past decade (Supplementary Fig. 1), in line with reduced AM sales^14^.

The AMs detected in our study largely reflect reported patterns of national use. Sulfaquinoxaline, sulfaclozine, sulfamethazine, and sulfadimethoxine are often administered for their anticoccidial activity^15^ and may not always be recognised by poultry workers as AM. Oxytetracycline and doxycycline are commonly used as first-line tetracyclines. The WP of most tilmicosin formulations is long (12 days), and this may explain the frequency of detection. The higher prevalence of non-compliance on farms compared with endpoints is consistent with additional days allowing WPs to elapse. Nonetheless, some very high residue levels at endpoints suggest possible deliberate administration shortly before sale or during transit to mask illness and enable marketing of sick birds.

Reporting of ongoing or recent AM use for non-compliant farm samples was more reliable in Bangladesh than in India or Vietnam. Root cause analysis (Supplementary Fig. 2) indicates that the most likely explanation is late administration of AMs combined with under-reporting. While inter-breed variations in pharmacokinetics and tissue depletion may contribute^16^, failure to observe adequate WPs remains the main cause of residue violation. Four additional scenarios could explain non-compliance in the absence of reported use: (i) off-label or unreported administration by farmers (e.g. stockpiling, reuse of leftover medicine)^17^; (ii) cross-contamination of supposedly non-medicated feed when produced on shared feedmill lines^18^; (iii) re-entry of AMs into the production cycle via feather meal, widely used as fertiliser or feed ingredient in Bangladesh and India^19,20^; and (iv) environmental contamination from persistent residues in chicken droppings^21^ or surface water and crops^22^.

Our results emphasise that all investigated farming systems/chicken types are affected and understanding the actual drivers in each system could yield better results than generic recommendations. From our experience in three countries, we recognise (i) limited awareness of which drugs are AM, (ii) low awareness of residues and resistance risks across the production system, and (iii) insufficient package labelling and guidance on withdrawal periods as actionable targets to lower non-compliance through strengthening control of usage, abandoning prophylactic use, conditioning AM usage to veterinary prescription and increasing stakeholder education^23^. Economic pressure and market instability have discouraged farmers from investing in biosecurity and complying fully with AM regulations. Residue concerns also result in non-tariff barriers to international trade. Finally, there is a need for reliable residue surveillance platforms. In resource-limited settings, cost-efficiency is typically achieved through validated microbiological screening methods (like the EU 4 Plate Test^24^ or the Five Plates Test^25^) first, followed by AM chromatographic identification for confirmation of suspect samples^15^. High investments and running costs, and reliance on specialised expertise, remain key barriers to broader implementation of chromatographic methods.

A limitation of this study is the absence of a second LC-MS/MS analysis with a quantitative method to confirm concentration. In the EU, all samples exceeding 0.5 MRL undergo this confirmatory testing. However, experience from the French national reference laboratory, which routinely applies this protocol, indicates that the screening assay rarely produces false positives. Here, we applied LC-MS/MS to all samples collected and all screened AMs corresponded to drugs routinely used in the three study countries.

MRLs for veterinary drugs vary across regions^23^. For poultry meat, the Codex Alimentarius^26^ and the US-FDA 21 CFR 556^27^ specify MRLs for only 13 and 23 veterinary medicines, respectively, compared with 49 in the EU. Adopting the EU MRLs permitted the most comprehensive analysis of our dataset. Comparing Codex Alimentarius MRLs directly with their equivalent EU MRLs found six were identical, six were higher (≤2x), and one was lower (1/2). Applying the Codex MRLs to our data, for these 13 AM, modestly reduced non-compliant prevalence from 5.4% (EU MRL) to 3.4% for all sites– primarily due to higher thresholds for tetracyclines and tilmicosin – but overall prevalence remains high.

Recommendations for policy implementations are context dependent and have been expressed in the national action plans on Antimicrobial Resistance in Bangladesh^28^, Vietnam^29–31^ and India^32^. Actionable plans are the strengthening conditions of prescription and delivery to a pool of actors that understand food residues and antimicrobial resistance and the limitation of over-the-counter sales. This requires stakeholder engagement and communication for behaviour change, with education of farmers and development of better package labelling to highlight withdrawal periods. Central initiatives are required to incentivise investments to strengthen biosecurity and stabilise market fluctuation. Enforcement mechanisms need to be in place to track excessive AMU and residue violations with an aim to reduce the incidence of domestically consumed meat to less than 1% across the production network evaluated herein. Finally, country-based and international One Health cross-sector collaboration is urgently needed to accelerate capacity building and the generation of evidence-guiding policies.

In conclusion, the high prevalence of AM residues above MRL in chicken meat represents a public health concern at both national and regional levels. It raises the urgent need for robust residue surveillance platforms to test products destined for local consumers in these and other countries where poultry production intensifies rapidly. Understanding the complex interplay between perceived need for AMs in existing farming practices, drug procurement chains, and the lack of disincentives to AM use requires a transdisciplinary approach. Identifying the source of residues and implementing stewardship measures, involving stakeholders beyond farmers alone, are key priorities to protect consumer health.

## Methods

### Ethical statement

Ethical approvals were obtained from the National Institute of Veterinary Research, Vietnam (020-433/DD-YTCC), Chattogram Veterinary and Animal Sciences University, Bangladesh (EC/2020/165/2/1) and the Royal Veterinary College Ethics Welfare Committee (URN:2020 1983-3, covering Gujarat). Each chicken purchased for sampling was culled ethically by trained staff using Schedule 1 approved methods (cervical dislocation).

Detailed methods are provided in Supplementary Information. This cross-sectional study targeted the most common chicken types in each zone. Endpoints were identified and selected through stratified cluster sampling (Fig. 1a). One chicken (occasionally two) of each type was sampled per endpoint, as well as in a farm randomly selected within the area supplying that endpoint. In farms, chickens were sampled towards the end of their production cycle.

Questionnaires (with informed consent) were completed on farms to document expected age and weight at sale, and use of AMs during production (Supplementary Fig. 3). At endpoints, the seller was asked to disclose any drug administration that occurred during transportation (Supplementary Fig. 4). Each chicken purchased for sampling was ethically culled, pectoralis muscle samples were immediately prepared, placed in a labelled plastic bag, then stored and shipped frozen until analysis. A semi-quantitative liquid chromatography tandem mass spectrometry (LC-MS/MS) method, adapted from the EU reference laboratory^10^ was used to screen 69 veterinary AM^9^ (validated in Hedges et al. 2024, summarised in Supplementary study protocol). The LC–MS/MS was validated in accordance with CRL 20/1/2010^33^ guidelines for the validation of screening methods for residues of veterinary medicines, ensuring <5% false negatives at the validation concentration (Cval). Cval (ranged between 12.5 and 400 µg/kg) was usually 50% of MRL and above the Limit of Detection. Recoveries were validated with sulfaphenazole as an internal standard.

A “non-compliant result” was defined as a detection above the EU maximum residue level (MRL), while a “non-compliant sample” contained one or more such results. For each non-compliant sample, we reported the number of non-compliant results, concentration category (>10xMRL, > 1xMRL), the AM(s) detected and their AMEG classification^5^.

We aimed to assess differences in residue prevalence across pre-defined strata of the study population, defined by variables central to the study design. Zone, site type, chicken type and finished status were therefore identified a priori as key explanatory variables for the logistic regression analysis^34,35^ (further justification in the Supplement). Separate model sets were specified for each of three datasets – combined farms and endpoints (chicken types sampled both on farms and at endpoints), farms only, and endpoints only. For each dataset, model formulations were adapted to the study design and data structure to avoid structural collinearity. In particular, all chickens sampled at endpoints were finished, and only a single chicken type was sampled on farms in Gujarat and Vietnam. For each dataset, we first fitted models to estimate associations between non-compliance and each key explanatory variable separately.

We then fitted models including all key explanatory variables simultaneously to estimate the independent association of each with non-compliance, while adjusting for the others. This approach allowed us to examine the robustness of results across alternative model formulations and how key system-level variables were associated with residue non-compliance. Time from sampling to testing was controlled for in all models. Additionally, we explored whether mean monthly temperature at the time of sampling and endpoint chicken weight (standardised for each zone and chicken type) were associated with non-compliance. Neither variable showed evidence of association with non-compliance, and their inclusion did not affect the estimated associations between non-compliance and the key explanatory variables (Supplementary Tables 5–7). Odds ratios (ORs) and 95% confidence intervals (CIs) were computed for each variable. Likelihood ratio tests were used to assess statistical support for all model terms; for categorical variables with >2 levels, level-specific *p*-values are reported for comparisons with the reference category. *P*-values below 0.05 were considered to provide statistical support for associations. All data management and analyses were performed using R^36^ (v4.2.1) and RStudio^37^ (v2022.7.1.554).

## Supplementary information

Supplementary information

## Data Availability

Full raw data about non-compliant samples are included in Supplementary Table 7. Researchers interested in accessing the data may contact the corresponding author with a detailed request.
