# Supplementary material for: High prevalence of antimicrobial residues in broiler meat in Bangladesh, India and Vietnam
Source: NPJ Sci Food. 2026 Apr 4;10:232. doi: 10.1038/s41538-026-00806-7 (PMC13421592; doi:10.1038/s41538-026-00806-7)
Supplement: Supplementary file 1 — Supplementary information [file 41538_2026_806_MOESM1_ESM.docx]

**npj Science of Food**

**Brief Communication**

**High prevalence of antimicrobial residues in broiler meat in Bangladesh, India and Vietnam**

**Supplementary information**

**Supplementary study protocol:**

***Study design***

This cross-sectional study targeted chicken types accounting for most the common form(s) chicken meat production in each zone (i.e. study area in a study region). These were fast-growing broilers in Gujarat, slow-growing broilers in northern Vietnam, and both fast- and slow-growing broilers in Bangladesh (Supplementary Table 1). Fast-growing broilers were commercial, white-feathered breeds such as Hubbard classic, Ross and Cobb500/Cobb430Y hybrids, slaughtered usually from 30-45 days of age, whereas slow-growing broilers were coloured-feather birds crossbred between commercial and/or local breeds raised for 55-80 days to several months (Sonali in Bangladesh). In each study region, the study area encompassed ≥4 administrative divisions (i.e. cities or provinces) and was defined to include a substantial fraction of the region through the areas supplying its *endpoints* –where chickens were slaughtered and/or sold to end-users –, thereby ensuring coverage of different agro-ecological zones.

The main types of endpoints were first identified in each zone. Endpoints were then selected by stratified cluster sampling, with stratification based on administrative divisions and/or their type. Depending on the local context, endpoint selection relied either on (i) sampling frames developed using existing datasets or through consultation of governmental organisations, or (ii) the generation of random spatial locations. Endpoints were live bird markets (referred to as markets) in Bangladesh, live bird shops (referred to as shops) in India, and both markets and slaughtering facilities in Vietnam. One chicken or occasionally two of each type were sampled per selected endpoint, as well as in farms randomly selected within the catchment area supplying that endpoint. As catchment areas were not formally defined and their characterisation was labour-intensive, a link-tracing approach was used to identify supplying farms. This approach, commonly used to study hard-to-reach populations, enabled the recruitment of farms within poultry supply networks while accounting for their underlying connectivity, as premises were selected through observed trading links.

In each zone, we aimed to sample 50 chickens per type at both farm and endpoint levels. In farms, chickens were sampled towards the end of their production cycle to offer greatest relevance to the likelihood of residue occurrence at their subsequent endpoint. Analysing a sample from a single bird per farm effectively detected antimicrobials (AM) residues, if present, in the flock [^1^](#_ENREF_1). An additional chicken type was selected in each zone and uniquely sampled in endpoints only: indigenous chickens in Bangladesh and India (Deshi) and fast-growing broilers in Vietnam. In Bangladesh and Vietnam, chickens were sampled, if present at the endpoints where the prioritised above mentioned chicken types were already sampled. In India, additional endpoints were selected with the aim of sampling 50 chickens of this additional type, one per endpoint.

***Ethical approvals***

Ethical approvals were obtained from the National Institute of Veterinary Research, Vietnam (020-433/DD-YTCC), Chattogram Veterinary and Animal Sciences University, Bangladesh (EC/2020/165/2/1) and the Royal Veterinary College Ethics Welfare Committee (URN:2020 1983-3, covering Gujarat).

***Sample collection and storage***

Sample collection in the three zones was conducted from March 2021 to April 2022. Maps of the locations in each zone were produced using GPS coordinates (Fig. 1a). Each chicken purchased for sampling was culled ethically by trained staff using approved methods. The samples were prepared immediately by removing the pectoralis muscle from the sternum to dissect a minimum of 20 g chicken muscle. Each sample was placed in a labelled plastic bag, stored at -20ºC during transportation and then stored at -80ºC until shipment (frozen) and storage at -80ºC until analysis.

***Questionnaire data on farms***

Questionnaires were completed on farms to assess husbandry practices, expected chicken age and weight at the time of sale, and the administration of AMs during the production cycle, together with the reason for AM administration (Supplementary Fig. 3). At endpoints, the seller was asked to disclose any drug administration that occurred during transportation (Supplementary Fig. 4). All participants who answered a questionnaire and/or provided birds for sampling gave their informed consent.

***Antimicrobial residue detection***

A liquid chromatography tandem mass spectrometry (LC-MS/MS) semi-quantitative analytical method was transferred from the EU reference laboratory [^2^](#_ENREF_2) and validated in the Singapore laboratory for 69 AMs [^1^](#_ENREF_1). Validation followed guidance from Council Directive (EC) 96/23, 29 April 1996 [^3^](#_ENREF_3). This has since been superseded by Regulation (EU) 2017/625 and its subsequent annexes, notably the Commission implementing Regulation (EU) 2021/808 has replaced the Commission Decision 2002/657 (EC) [^4^](#_ENREF_4) concerning the performance of analytical methods. The LC–MS/MS was validated according to CRL 20/1/2010[^5^](#_ENREF_5) guidelines for the validation of screening methods for residues of veterinary medicines to ensure that there were no more than 5% false negatives at the Cval, the validation concentration which was usually 50% of MRL and superior to the Limit of Detection. Azithromycin was added to the panel and validated at 50 ug/kg (as there is no maximum residue levels MRL defined for azithromycin, its detection level was aligned with the MRL of tilmicosin). In brief, analytical standards were purchased as analytical grade reagents to prepare individual standard stock solutions. Two pools of spiking solutions were prepared: pool A contained 37 analytes from beta lactam, sulfonamide and tetracyclines families, and pool B contained 31 analytes from the quinolone, amphenicol, macrolide, and pleuromutilin families, as well as miscellaneous antibiotics, including trimethoprim (Table below). Each chicken muscle sample (minimum 20 grams) was minced in a food chopper (La Moulinette, Tefal). Spiked control samples with pool A or pool B analytes (QCA and QCB) were prepared to the targeted concentration in advance and frozen to at least -18°C for a validated maximum storage duration of 6 months. The internal standard sulfaphenazole (1 µg/mL; Sigma-Aldrich) was added to tubes containing 2 g of the minced meat samples before liquid extraction with acetonitrile and evaporation of the supernatant under a nitrogen stream at 30ºC. The residue was reconstituted in mobile phase and filtered before injection in the chromatographic column of an HPLC machine (ACQUITY UPLC I-Class PLUS Waters Pacific Pte Ltd) connected to a triple quadrupole mass spectrometer (Xevo TQ-S, Waters, Singapore). The mass spectrometer was operated in positive electrospray ionisation mode (ESI+), using multiple reaction monitoring (MRM) detections to screen the presence of 69 AMs characterised by their retention time and their two strongest compound transitions (Transitions Tr1 and Tr2) (Table below). A blank sample control and the two spiked controls were run for every ~10 samples to monitor method performance.

A positive sample was defined by the presence of at least one AM at concentration ≥ MRL. A “non-compliant result” corresponds to the detection of an AM at a concentration equal or above the MRL. A “non-compliant sample” was a sample with one or more non-compliant result.

**Supplementary Table 1: Retention times (RT), Validation concentration (CVal, µg/kg) MRM transitions (Precursor ion> Product ions with Transition 1 and Transition 2), and mass spectrometer conditions used for each compound for veterinary antimicrobial residue detection by liquid chromatography tandem mass spectrometry (LC-MS/MS)**.

| **Analyte** | **CVal**  **(µg/kg)** | **Transition** | **RT (min)** | **Precursor ion (m/z)2** | **Product ion (m/z)*** | **Cone voltage** | **Collision Energy** | **Dwell time (ms)** |
| --- | --- | --- | --- | --- | --- | --- | --- | --- |
| **Sulfaguanidine** | 100 | Tr1 | 2.03 | 215.0 | **156.0** | 30 | 14 | 25 |
|  |  | Tr2 |  | 215.0 | **108.0** | 30 | 20 | 25 |
| **Florfenicol amine** | 50 | Tr1 | 2.84 | 248.1 | **230.0** | 20 | 10 | 25 |
|  |  | Tr2 |  | 248.1 | **130.1** | 20 | 22 | 25 |
| **Sulfadiazine** | 50 | Tr1 | 2.96 | 251.0 | **108.1** | 52 | 20 | 25 |
|  |  | Tr2 |  | 251.0 | **156.0** | 52 | 12 | 25 |
| **Amoxicillin** | 50 | Tr1 | 3.38 | 366.1 | **349.0** | 14 | 8 | 25 |
|  |  | Tr2 |  | 366.1 | **208.0** | 14 | 10 | 25 |
| **Sulfacetamide** | 50 | Tr1 | 2.86 | 215.1 | **156.0** | 30 | 10 | 29 |
|  |  | Tr2 |  | 215.1 | **108.0** | 30 | 15 | 29 |
| **Sulfathiazole** | 50 | Tr1 | 3.26 | 256.0 | **156.0** | 58 | 14 | 25 |
|  |  | Tr2 |  | 256.0 | **108.1** | 58 | 22 | 25 |
| **Sulfamerazine** | 50 | Tr1 | 3.38 | 265.0 | **156.0** | 60 | 14 | 25 |
|  |  | Tr2 |  | 265.0 | **108.1** | 60 | 26 | 25 |
| **Desacetyl cephapirin** | 50 | Tr1 | 2.44 | 382.0 | **112.0** | 40 | 22 | 25 |
|  |  | Tr2 |  | 382.0 | **152.0** | 40 | 24 | 25 |
| **Cephapirin** | 50 | Tr1 | 3.68 | 424.1 | 291.9 | 16 | 14 | 25 |
|  |  | Tr2 |  | 424.1 | **151.9** | 16 | 22 | 25 |
| **Cefquinome** | 25 | Tr1 | 3.91 | 529.4 | **134.0** | 34 | 12 | 25 |
|  |  | Tr2 |  | 529.4 | **125.0** | 34 | 52 | 25 |
| **Sulfadimerazine** | 50 | Tr1 | 3.73 | 279.1 | 186.1 | 56 | 16 | 25 |
|  |  | Tr2 |  | 279.1 | **156.0** | 56 | 18 | 25 |
| **Lincomycin** | 50 | Tr1 | 4.01 | 407.1 | **126.0** | 86 | 14 | 25 |
|  |  | Tr2 |  | 407.1 | **359.1** | 86 | 18 | 25 |
| **Cephalonium** | 25 | Tr1 | 4.06 | 459.0 | **152.0** | 12 | 16 | 25 |
|  |  | Tr2 |  | 459.0 | **337.0** | 12 | 8 | 25 |
| **Sulfamethoxypyridazine** | 50 | Tr1 | 4.17 | 281.2 | **156.0** | 58 | 16 | 25 |
|  |  | Tr2 |  | 281.2 | **108.1** | 58 | 22 | 25 |
| **Cephalexin** | 100 | Tr1 | 4.60 | 348.0 | **158.0** | 2 | 6 | 25 |
|  |  | Tr2 |  | 348.0 | 174.0 | 2 | 14 | 25 |
| **Ampicillin** | 25 | Tr1 | 4.53 | 350.1 | **106.0** | 2 | 16 | 25 |
|  |  | Tr2 |  | 350.1 | **160.1** | 2 | 10 | 25 |
| **Marbofloxacin** | 75 | Tr1 | 4.55 | 363.1 | **345.0** | 20 | 26 | 25 |
|  |  | Tr2 |  | 363.1 | **320.0** | 20 | 14 | 25 |
| **Norfloxacin** | 50 | Tr1 | 4.61 | 320.1 | **302.0** | 66 | 20 | 25 |
|  |  | Tr2 |  | 320.1 | **230.9** | 66 | 38 | 25 |
| **Thiamphenicol** | 25 | Tr1 | 3.94 | 356.1 | **307.9** | 4 | 14 | 25 |
|  |  | Tr2 |  | 358.0 | **310.0** | 4 | 14 | 25 |
| **Sulfamethisole** | 50 | Tr1 | 4.20 | 271.0 | **156.0** | 56 | 14 | 25 |
|  |  | Tr2 |  | 271.0 | **108.1** | 56 | 24 | 25 |
| **Trimethoprim** | 25 | Tr1 | 4.73 | 291.1 | **123.1** | 30 | 24 | 25 |
|  |  | Tr2 |  | 291.1 | **230.1** | 30 | 20 | 25 |
| **Ciprofloxacin** | 25 | Tr1 | 4.75 | 332.1 | **314.0** | 2 | 20 | 25 |
|  |  | Tr2 |  | 332.1 | **230.9** | 2 | 36 | 25 |
| **Oxytetracycline** | 50 | Tr1 | 4.75 | 461.1 | **426.1** | 4 | 18 | 25 |
|  |  | Tr2 |  | 461.1 | **443.0** | 4 | 12 | 25 |
| **Tildipirosin** | 200 | Tr1 | 5.42 | 734.3 | **98.0** | 30 | 36 | 25 |
|  |  | Tr2 |  | 734.3 | **174.0** | 30 | 36 | 25 |
| **Danofloxacin** | 100 | Tr1 | 4.85 | 358.0 | **340.0** | 42 | 20 | 25 |
|  |  | Tr2 |  | 358.0 | **255.0** | 42 | 34 | 25 |
| **Cefazolin** | 25 | Tr1 | 4.42 | 455.2 | **322.9** | 2 | 10 | 25 |
|  |  | Tr2 |  | 455.2 | **155.9** | 2 | 20 | 25 |
| **Enrofloxacin** | 25 | Tr1 | 5.11 | 360.0 | **342.0** | 2 | 20 | 25 |
|  |  | Tr2 |  | 360.0 | **286.0** | 2 | 32 | 25 |
| **Tetracycline** | 50 | Tr1 | 5.19 | 445.0 | **410.0** | 2 | 18 | 25 |
|  |  | Tr2 |  | 445.0 | 154.0 | 2 | 26 | 25 |
| **Neospiramycin** | 50 | Tr1 | 5.94 | 699.4 | 174.0 | 56 | 28 | 35 |
|  |  | Tr2 |  | 699.4 | 540.1 | 56 | 18 | 35 |
| **Sulfamonomethoxine** | 50 | Tr1 | 4.69 | 281.1 | **156.0** | 36 | 16 | 25 |
|  |  | Tr2 |  | 281.1 | 108.0 | 36 | 26 | 25 |
| **Tulathromycin** | 400 | Tr1 | 6.28 | 806.4 | **577.2** | 82 | 20 | 25 |
|  |  | Tr2 |  | 806.4 | **158.0** | 82 | 38 | 25 |
| **Sarafloxacin** | 15 | Tr1 | 5.68 | 386.0 | **368.0** | 2 | 20 | 25 |
|  |  | Tr2 |  | 386.0 | 342.0 | 2 | 18 | 25 |
| **Difloxacin** | 150 | Tr1 | 5.82 | 400.0 | **356.0** | 2 | 20 | 25 |
|  |  | Tr2 |  | 400.0 | **382.0** | 2 | 24 | 25 |
| **Cefoperazone** | 25 | Tr1 | 5.27 | 646.4 | **143.0** | 22 | 34 | 25 |
|  |  | Tr2 |  | 646.4 | **530.0** | 22 | 10 | 25 |
| **Spiramycin** | 50 | Tr1 | 6.65 | 843.4 | **174.0** | 60 | 36 | 28 |
|  |  | Tr2 |  | 843.4 | 142.0 | 60 | 34 | 28 |
| **Dapsone** | 25 | Tr1 | 4.92 | 249.0 | **156.0** | 40 | 12 | 25 |
|  |  | Tr2 |  | 249.0 | **108.0** | 40 | 20 | 25 |
| **Cefuroxime** | 25 | Tr1 | 4.92 | 447.1 | **385.9** | 16 | 12 | 25 |
|  |  | Tr2 |  | 447.1 | **341.9** | 16 | 12 | 25 |
| **Sulfachloropyridazine** | 50 | Tr1 | 5.05 | 285.0 | **156.0** | 18 | 14 | 25 |
|  |  | Tr2 |  | 285.0 | **108.1** | 18 | 26 | 25 |
| **Sulfadoxine** | 50 | Tr1 | 5.27 | 311.1 | **156.0** | 2 | 16 | 25 |
|  |  | Tr2 |  | 311.1 | **108.0** | 2 | 24 | 25 |
| **Chlortetracycline** | 50 | Tr1 | 6.57 | 479.0 | **444.1** | 10 | 20 | 25 |
|  |  | Tr2 |  | 479.0 | **462.0** | 10 | 16 | 25 |
| **Doxycycline** | 50 | Tr1 | 6.88 | 445.0 | **428.0** | 2 | 18 | 25 |
|  |  | Tr2 |  | 445.0 | **154.0** | 2 | 30 | 25 |
| **Ceftiofur** | 50 | Tr1 | 6.81 | 524.0 | **240.9** | 6 | 16 | 25 |
|  |  | Tr2 |  | 524.0 | **125.1** | 6 | 58 | 25 |
| **Gamithromycin** | 25 | Tr1 | 7.05 | 777.5 | **619.3** | 60 | 28 | 28 |
|  |  | Tr2 |  | 777.5 | **158.0** | 60 | 35 | 28 |
| **Tilmicosin** | 25 | Tr1 | 7.13 | 869.7 | **174.1** | 58 | 40 | 25 |
|  |  | Tr2 |  | 869.7 | **696.4** | 58 | 40 | 25 |
| **Erythromycin** | 100 | Tr1 | 7.25 | 734.5 | **158.1** | 30 | 30 | 35 |
|  |  | Tr2 |  | 734.5 | **576.4** | 30 | 20 | 35 |
| **Tylosin** | 50 | Tr1 | 7.27 | 916.4 | **174.2** | 60 | 35 | 25 |
|  |  | Tr2 |  | 916.4 | **772.5** | 60 | 30 | 25 |
| **Sulfamethoxazole** | 50 | Tr1 | 5.56 | 254.0 | **156.0** | 46 | 16 | 25 |
|  |  | Tr2 |  | 254.0 | **108.0** | 46 | 24 | 25 |
| **O-acetyltylosin** | 12.5 | Tr1 | 7.40 | 958.7 | **174.2** | 60 | 35 | 19 |
|  |  | Tr2 |  | 958.7 | **814.5** | 60 | 30 | 19 |
| **Oxolinic acid** | 50 | Tr1 | 6.09 | 262.1 | **244.0** | 56 | 20 | 25 |
|  |  | Tr2 |  | 262.1 | **215.9** | 56 | 24 | 25 |
| **Florfenicol** | 50 | Tr1 | 5.59 | 358.0 | 241.0 | 12 | 16 | 25 |
|  |  | Tr2 |  | 360.0 | 243.0 | 12 | 16 | 25 |
| **Sulfaquinoxaline** | 50 | Tr1 | 6.75 | 301.1 | **156.0** | 30 | 18 | 25 |
|  |  | Tr2 |  | 301.1 | **108.0** | 30 | 26 | 25 |
| **Tiamulin** | 50 | Tr1 | 7.72 | 494.3 | **192.0** | 28 | 16 | 25 |
|  |  | Tr2 |  | 494.3 | **119.0** | 28 | 40 | 25 |
| **Sulfaclozine** | 50 | Tr1 | 6.72 | 285.0 | 155.9 | 2 | 16 | 25 |
|  |  | Tr2 |  | 285.0 | **130.1** | 2 | 20 | 25 |
| **Sulfadimethoxine** | 50 | Tr1 | 6.75 | 311.1 | **156.0** | 2 | 20 | 25 |
|  |  | Tr2 |  | 311.1 | **108.0** | 2 | 30 | 25 |
| **Valnemulin** | 25 | Tr1 | 7.83 | 565.4 | **263.1** | 30 | 16 | 19 |
|  |  | Tr2 |  | 565.4 | **147.1** | 30 | 36 | 19 |
| **Josamycin** | 25 | Tr1 | 7.88 | 828.5 | **174.2** | 12 | 30 | 25 |
|  |  | Tr2 |  | 828.5 | 109.1 | 12 | 48 | 25 |
| **Sulfaphenazole** | 50 | Tr1 | 6.93 | 315.0 | 156.0 | 32 | 20 | 28 |
|  |  | Tr2 |  | 315.0 | 158.0 | 32 | 28 | 28 |
| **Penicillin G** | 25 | Tr1 | 7.30 | 335.0 | **159.9** | 28 | 16 | 25 |
|  |  | Tr2 |  | 335.0 | **176.0** | 28 | 20 | 25 |
| **Tylvalosin** | 12.5 | Tr1 | 8.11 | 1042.8 | 109.1 | 60 | 45 | 60 |
|  |  | Tr2 |  | 1042.8 | 174.2 | 60 | 38 | 60 |
| **Penicillin V** | 25 | Tr1 | 7.58 | 351.1 | **160.0** | 28 | 12 | 35 |
|  |  | Tr2 |  | 351.1 | **114.0** | 28 | 32 | 35 |
| **Nalidixic acid** | 25 | Tr1 | 7.35 | 233.1 | **186.9** | 50 | 26 | 25 |
|  |  | Tr2 |  | 233.1 | **215.0** | 50 | 20 | 25 |
| **Oxacillin** | 150 | Tr1 | 7.74 | 402.1 | **160.0** | 4 | 14 | 25 |
|  |  | Tr2 |  | 402.1 | **243.0** | 4 | 14 | 25 |
| **Flumequine** | 100 | Tr1 | 7.53 | 262.0 | **244.0** | 50 | 20 | 25 |
|  |  | Tr2 |  | 262.0 | **201.9** | 50 | 32 | 25 |
| **Nafcillin** | 150 | Tr1 | 8.03 | 415.0 | **199.1** | 6 | 18 | 25 |
|  |  | Tr2 |  | 415.0 | **171.1** | 6 | 34 | 25 |
| **Cloxacillin** | 150 | Tr1 | 7.94 | 436.0 | **160.1** | 2 | 10 | 25 |
|  |  | Tr2 |  | 436.0 | **277.0** | 2 | 10 | 25 |
| **Dicloxacillin** | 150 | Tr1 | 8.21 | 470.3 | **160.0** | 22 | 10 | 25 |
|  |  | Tr2 |  | 470.3 | **310.8** | 22 | 15 | 25 |
| **Rifaximin** | 25 | Tr1 | 8.34 | 786.5 | **754.5** | 22 | 20 | 25 |
|  |  | Tr2 |  | 786.5 | **151.1** | 22 | 38 | 25 |
| **Novobiocin** | 25 | Tr1 | 9.05 | 613.2 | **189.1** | 52 | 34 | 25 |
|  |  | Tr2 |  | 613.2 | **218.1** | 52 | 12 | 25 |
| **Azithromycin** | 50 | Tr1 | 6.81 | 749.3 | **591.4** | 24 | 24 | 25 |
|  |  | Tr2 |  | 749.3 | **158.1** | 24 | 34 | 25 |

***Sample analysis and LC-MS/MS data post-processing***

After extraction (July 2021 to August 2022 for Vietnam, August 2022 to November 2022 for Bangladesh and India), samples were analysed in batches of 18 to 57 samples. Raw peak Area Under the Curve and retention times data were quality-assessed and evaluated both manually (Excel workflow) and automatically through a R script, following a bespoke decision algorithm [^1^](#_ENREF_1). A “non-compliant result” corresponded to the detection of an AM at a concentration equal or above the MRL. A “non-compliant sample” was a sample with one or more non-compliant results. For each non-compliant sample, we reported the number of non-compliant results, the concentration range (> 10 MRL if more than 10 times MRL, > 1 MRL otherwise), the AM identified and the AMEG category they belong to [^6^](#_ENREF_6), the chicken weight, and, when available on farm and/ or endpoints, the age of the chicken and the treatment history.

**Statistical analysis**

Mean monthly temperatures were obtained from data publicly available online (World Bank, n.d., “Gujarat, IN Climate Zone, Monthly Weather Averages and Historical Data,” n.d.). All other data were extracted from questionnaires and observations from field sampling. A chicken was considered “finished” if either the current age or weight were superior or equal to the expected age and weight at sale, and “not finished” if current age and weight were inferior to expected age and weight at sale. Where this information was missing for a chicken, we assumed it was inferior to the expected age or weight at sale.

Mean monthly temperatures was included as more AM are used by farmers in the rainy / cold season, based on their previous experience. The Zone variable corresponds to the country or location within country as local policies relating to the dispensing and the use of AMD and residue control differ between zones. Site (farm vs endpoint) was included as the meat residue from the last treatment administered on farm fades away (below MRL) as time passed during chicken transportation to the endpoint. The “chicken type” variable explored whether fast growing broilers (intensive production) or slow growing broilers (extensive production) systems require different antimicrobials use. The “Finished” status us a binary variable, on farm only, to flag when farm sampling may not have occurred with certainty at the end of the production period, as not having reached the weight or age expected as sale. By definition, the status of the bird was “Finished” when a chicken was collected from an endpoint. Time from sampling to testing was included in the model as limited degradation of AMD during storage when exceeding 6 months at -80⁰C.

**Supplementary Table 2: Number of samples collected from each country, type of site, type of chickens (including visits where several samples were collected):**

| **Zone** | **Site type** | **Bird type** | **Total number of sites** | **Total number of samples** | **Number of endpoint visits where 1 to 6 independent samples were collected** | | | | | |
| --- | --- | --- | --- | --- | --- | --- | --- | --- | --- | --- |
|  |  |  |  |  | 1 sample | 2 samples | 3 samples | 4 samples | 5 samples | 6 samples |
| All | Farm | All | 195 | 195 | 195 | - | - | - | - | - |
| All | Endpoint | All | 166 | 363 | 64 | 45 | 29 | 19 | 8 | 1 |
|  |  |  |  |  |  |  |  |  |  |  |
| Bangladesh | Farm | All | 99 | 99 | 99 | - | - | - | - | - |
| Bangladesh | Farm | Fast-growing | 49 | 49 | 49 | - | - | - | - | - |
| Bangladesh | Farm | Slow-growing | 50 | 50 | 50 | - | - | - | - | - |
| Bangladesh | Endpoint | All | 50 | 174 | 0 | 3 | 25 | 18 | 3 | 1 |
| Bangladesh | Endpoint | Fast-growing | 49 | 54 | 44 | 5 | - | - | - | - |
| Bangladesh | Endpoint | Slow-growing | 50 | 59 | 41 | 9 | - | - | - | - |
| Bangladesh | Endpoint | Indigenous | 47 | 61 | 33 | 14 | - | - | - | - |
| Gujarat (India) | Farm | Fast-growing | 50 | 50 | 50 | - | - | - | - | - |
| Gujarat (India) | Endpoint | All | 65 | 108 | 28 | 32 | 4 | 1 | - | - |
| Gujarat (India) | Endpoint | Fast-growing | 50 | 51 | 49 | 1 | - | - | - | - |
| Gujarat (India) | Endpoint | Indigenous | 50 | 57 | 43 | 7 | - | - | - | - |
| Vietnam | Farm | Slow-growing | 46 | 46 | 46 | - | - | - | - | - |
| Vietnam | Endpoint | All | 51 | 81 | 36 | 10 | - | - | 5 | - |
| Vietnam | Endpoint | Fast-growing | 12 | 16 | 11 | - | - | - | 1 | - |
| Vietnam | Endpoint | Slow-growing | 49 | 65 | 45 | - | - | - | 4 | - |

Legend: fast-growing broilers: white feathered breeds, slow-growing broilers = coloured feather crossbreds i.e. "hybrids" in Vietnam and Sonali in Bangladesh. Indigenous chickens: Deshi in Bangladesh and India. Endpoint: either live bird market (Bangladesh, Vietnam), Live bird shop (Gujarat) or slaughtering facility (Vietnam).

**Supplementary Table 3: Breeds of broiler chicken in the three zones:**

| Zone | Site | Fast-growing broilers breeds | Slow-growing broilers or indigenous chickens |
| --- | --- | --- | --- |
| Bangladesh | Farms | Cobb500, Hybro-PN, Hubbard classic, Ross. | Sonali* |
|  | Endpoints |  | Deshi, Sonali* |
| Gujarat (India) | Farms | Cobb430Y, Sunbro | Farm breeds: Deshi (desi) |
|  | Endpoints | Cobb400 | - |
| Vietnam | Farms and Endpoints | Arbor Acres | Hybrid of the Ho, Choi, Ri or Mia breeds**  Fayoumi chicken |

* Sonali: cross between Fayoumi Hen and Rhode Island Red Cock in Bangladesh, **Ho, Choi, Ri and Mia are Indigenous breeds in Vietnam.

**Supplementary Table 4: Sizes of the farms visited:**

| **Location** | **Type of sites** | **N** | **Size proxy** | **Median size (25 – 75^th^ percentile)** |
| --- | --- | --- | --- | --- |
| Bangladesh | Farms | 99 | Maximum capacity of birds | 2500 (1200-4000) |
| Gujarat (India) | Farms | 50 | Maximum capacity of birds | 9500 (5000-13500) |
| Vietnam | Farms | 46 | Maximum capacity of birds | 4000 (1375-5750) |

N: number of facilities

**Supplementary Table 5.: Joint farm-endpoint logistic regression model:**

| **Variable** | **n** | **non-compliant**  **(%)** | **Set 1**  **OR (95% CI)** | ***P*** | **Set 2**  **OR (95% CI)** | ***P*** | **Set 3**  **OR (95% CI)** | ***P*** |
| --- | --- | --- | --- | --- | --- | --- | --- | --- |
| Zone-chicken type |  |  |  |  |  |  |  |  |
| Bangladesh - fast-growing broilers | 103 | 18 (17.5%) | 1 | 0.063 | 1 | 0.133 | 1 | 0.114 |
| Bangladesh - slow-growing broilers | 109 | 18 (16.5%) | 0.90 (0.44-1.86) | *0.777* | 0.87 (0.41-1.82) | *0.705* | 0.88 (0.41-1.84) | *0.725* |
| Gujarat - fast-growing broilers | 101 | 9 (8.9%) | 0.39 (0.16-0.92) | *0.032* | 0.43 (0.16-1.09) | *0.075* | 0.38 (0.13-1.04) | *0.060* |
| Vietnam - slow-growing broilers | 111 | 13 (11.7%) | 0.28 (0.07-0.93) | *0.038* | 0.28 (0.07-0.98) | *0.046* | 0.28 (0.07-0.98) | *0.047* |
| Finished-site |  |  |  |  |  |  |  |  |
| Finished-endpoint | 229 | 19 (8.3%) | 1 | 0.001 | 1 | 0.001 | 1 | 0.001 |
| Finished-farm | 107 | 17 (15.9%) | 2.08 (1.02-4.19) | *0.043* | 2.30 (1.10-4.79) | *0.027* | 2.36 (1.12-4.96) | *0.024* |
| Unfinished-farm | 88 | 22 (25.0%) | 3.75 (1.91-7.45) | *<0.001* | 3.37 (1.68-6.85) | *0.001* | 3.34 (1.66-6.79) | *0.001* |
| Time from sampling to testing (unit: 30 days) |  |  | - |  | 0.84 (0.68-1.02) | 0.073 | 0.83 (0.68-1.01) | 0.061 |
| Mean monthly temperature at sampling (°C) |  |  | - |  | - |  | 1.03 (0.94-1.14) | 0.538 |

Set 1: models for each key explanatory variable individually while controlling for the time from sampling to testing; Set 2: model for all key explanatory variables while controlling for the time from sampling; Set 3: Set 2 model with mean monthly temperature at sampling; Finished vs Unfinished chicken: when either the current age or weight were superior or equal to the expected age and expected weight at sale; OR: odds ratio; CI: Confidence Interval; *P*: P-value; italicised P-values correspond to likelihood ratio tests assessing the statistical support for each variable, whereas non-italicised P-values (for categorical variables with >2 levels) correspond to level-specific comparisons with the reference category.

**Supplementary Table 6: Farm logistic regression model:**

| **Variable** | **n** | **non-compliant**  **(%)** | **Set 1**  **OR (95% CI)** | ***P*** | **Set 2**  **OR (95% CI)** | ***P*** | **Set 3**  **OR (95% CI)** | ***P*** |
| --- | --- | --- | --- | --- | --- | --- | --- | --- |
| Zone-chicken type |  |  |  |  |  |  |  |  |
| Bangladesh - fast-growing broilers | 49 | 14 (28.6%) | 1 | 0.125 | 1 | 0.199 | 1 | 0.179 |
| Bangladesh - slow-growing broilers | 50 | 11 (22.0%) | 0.64 (0.25-1.61) | *0.346* | 0.59 (0.22-1.50) | *0.267* | 0.60 (0.23-1.53) | *0.286* |
| Gujarat - fast-growing broilers | 50 | 7 (14.0%) | 0.33 (0.11-0.94) | *0.037* | 0.44 (0.13-1.44) | *0.174* | 0.37 (0.10-1.34) | *0.129* |
| Vietnam - slow-growing broilers | 46 | 7 (15.2%) | 0.19 (0.03-0.96) | *0.045* | 0.18 (0.03-0.91) | *0.038* | 0.18 (0.03-0.92) | *0.039* |
| Finished chicken |  |  |  |  |  |  |  |  |
| No | 88 | 22 (25.0%) | 1 | 0.119 | 1 | 0.244 | 1 | 0.315 |
| Yes | 107 | 17 (15.9%) | 0.57 (0.27-1.16) | - | 0.60 (0.24-1.41) | - | 0.63 (0.25-1.53) | - |
| Time from sampling to testing (unit: 30 days) |  |  | - |  | 0.82 (0.61-1.06) | 0.135 | 0.80 (0.60-1.05) | 0.111 |
| Mean monthly temperature at sampling (°C) |  |  | - |  | - |  | 1.04 (0.92-1.18) | 0.495 |

Set 1: models for each key explanatory variable individually while controlling for the time from sampling to testing; Set 2: model for all key explanatory variables while controlling for the time from sampling; Set 3: Set 2 model with mean monthly temperature at sampling; Finished vs Unfinished chicken: when either the current age or weight were superior or equal to the expected age and expected weight at sale; OR: odds ratio; CI: Confidence Interval; *P*: P-value; italicised P-values correspond to likelihood ratio tests assessing the statistical support for each variable, whereas non-italicised P-values (for categorical variables with >2 levels) correspond to level-specific comparisons with the reference category.

**Supplementary Table 7: Endpoint logistic regression model:**

| **Variable** | **n** | **non-compliant**  **(%)** | **Set 1**  **OR (95% CI)** | ***P*** | **Set 2**  **OR (95% CI)** | ***P*** | **Set 3**  **OR (95% CI)** | ***P*** | **Set 4**  **OR (95% CI)** | ***P*** |
| --- | --- | --- | --- | --- | --- | --- | --- | --- | --- | --- |
| Zone |  |  |  |  |  |  |  |  |  |  |
| Bangladesh | 174 | 15 (8.6%) | 1 | 0.103 | 1 | 0.139 | 1 | 0.180 | 1 | 0.149 |
| Gujarat | 108 | 4 (3.7%) | 0.32 (0.09-0.96) | *0.042* | 0.41 (0.10-1.34) | *0.143* | 0.45 (0.11-1.63) | *0.232* | 0.41 (0.10-1.36) | *0.148* |
| Vietnam | 81 | 7 (8.6 %) | 0.31 (0.05-1.54) | *0.158* | 0.24 (0.04-1.29) | *0.099* | 0.25 (0.04-1.29) | *0.102* | 0.25 (0.04-1.33) | *0.108* |
| Chicken type |  |  |  |  |  |  |  |  |  |  |
| Fast-growing broilers | 121 | 7 (5.8%) | 1 | 0.399 | 1 | 0.541 | 1 | 0.537 | 1 | 0.538 |
| Slow-growing broilers | 124 | 13 (10.5%) | 1.75 (0.66-4.94) | *0.263* | 1.64 (0.58-5.08) | *0.357* | 1.65 (0.58-5.09) | *0.355* | 1.65 (0.58-5.12) | *0.353* |
| Indigenous chickens | 118 | 6 (5.1%) | 0.91 (0.28-2.84) | *0.870* | 0.90 (0.28-2.83) | *0.854* | 0.90 (0.28-2.83) | *0.851* | 0.90 (0.28-2.85) | *0.859* |
| Time from sampling to testing (unit: 30 days) |  |  | - |  | 0.80 (0.61-1.03) | 0.083 | 0.81 (0.61-1.05) | 0.114 | 0.81 (0.61-1.03) | 0.092 |
| Mean monthly temperature at sampling (°C) |  |  | - |  | - |  | 0.97 (0.85-1.11) | 0.634 | - |  |
| Standardised chicken weight |  |  | - |  | - |  | - |  | 1.11 (0.74-1.62) | 0.614 |

Set 1: models for each key explanatory variable individually while controlling for the time from sampling to testing; Set 2: model for all key explanatory variables while controlling for the time from sampling; Set 3: Set 2 model with mean monthly temperature at sampling; Set 4: Set 2 model with mean monthly temperature at sampling; OR: odds ratio; CI: Confidence Interval; *P*: P-value; italicised P-values correspond to likelihood ratio tests assessing the statistical support for each variable, whereas non-italicised P-values (for categorical variables with >2 levels) correspond to level-specific comparisons with the reference category.

**Supplementary Table 8: List of non-compliant samples by country and site type with number of offending residues, level of highest residue, chicken characteristics and declared antimicrobial use. The name of the AM is followed by the number of days since the last reported administration (duration in days)**

| **Bangladesh** | **Number of AM > 1 MRL** | **Level > MRL for highest concentration** | **Breed** | **Site type** | **Weight (kg)** | **Age (d)** | **Finished**  **(Y/N)** | **Reported Antimicrobial Use** |
| --- | --- | --- | --- | --- | --- | --- | --- | --- |
| **Markets** |  |  |  |  |  |  |  |  |
| Tilmicosin | 1 | > 10 MRL | Sonali | Market | 0.75 |  | Y | no treatment reported |
| Tilmicosin | 1 | > 1 MRL | Sonali | Market | 0.79 |  | Y | no treatment reported |
| Tilmicosin | 1 | > 1 MRL | Sonali | Market | 0.81 |  | Y | no treatment reported |
| Tilmicosin | 1 | > 1 MRL | Sonali | Market | 1.00 |  | Y | no treatment reported |
| Tilmicosin | 1 | > 1 MRL | Sonali | Market | 1.04 |  | Y | no treatment reported |
| Tilmicosin | 1 | > 1 MRL | Sonali | Market | 0.67 |  | Y | no treatment reported |
| Tilmicosin | 1 | > 1 MRL | F-G Broil | Market | 2.1 |  | Y | no treatment reported |
| Tilmicosin | 1 | > 1 MRL | F-G Broil | Market | 1.3 |  | Y | no treatment reported |
| Oxytetracycline | 1 | > 10 MRL | Deshi | Market | 1.1 |  | Y | no treatment reported |
| Oxytetracycline | 1 | > 1 MRL | Deshi | Market | 0.96 |  | Y | no treatment reported |
| Oxytetracycline | 1 | > 1 MRL | Deshi | Market | 1.1 |  | Y | no treatment reported |
| Oxytetracycline | 1 | > 1 MRL | F-G Broil | Market | 1.5 |  | Y | no treatment reported |
| Oxytetracycline and Doxycycline | 2 | > 1 MRL | F-G Broil | Market | 1.7 |  | Y | no treatment reported |
| Doxycycline | 1 | > 1 MRL | F-G Broil | Market | 1.5 |  | Y | no treatment reported |
| Tetracycline | 1 | > 1 MRL | Deshi | Market | 1.1 |  | Y | no treatment reported |
| **Farms fast-growing broilers** |  |  |  |  |  |  |  |  |
| Sulfadiazine/trimethoprim | 2 | > 10 MRL | F-G Broil | Farm | 1.6 | 32 | N | none |
| Doxycycline*, Sulfadiazine* | 2 | > 1 MRL | F-G Broil | Farm | 2.2 | 30 | Y | colistin last 20d, 5d, oxytetracycline last 9d, 7d, [doxycycline, erythromycin, sulfadiazine, trimethoprim] now, 7d |
| Sulfamethazine, Sulfadimethoxine | 2 | > 10 MRL | F-G Broil | Farm | 1.7 | 29 | N | amoxicillin last 7d, 4d |
| Ciprofloxacin* | 1 | > 1 MRL | F-G Broil | Farm | 1.5 | 28 | N | ciprofloxacin now, 7d, colistin now, 3d |
| Doxycycline*, Sulfamethoxazole/trimethoprim | 3 | > 10 MRL | F-G Broil | Farm | 1.8 | 27 | N | amoxicillin last 8d, 7d, doxycycline now, 3d |
| Doxycycline*, Sulfadiazine*/trimethoprim* | 3 | > 10 MRL | F-G Broil | Farm | 1.1 | 25 | N | cefalexin last 5d, 5d, enrofloxacin last 5d, 5d, [doxycycline, erythromycin, sulfadiazine, trimethoprim] now, 2d |
| Norfloxacin* (pefloxacin metabolite) | 1 | > 1 MRL | F-G Broil | Farm | 1.5 | 30 | N | amoxicillin last 23d, 5d + last 16d, 3d + last 10d, 4d, ciprofloxacin last 3d, 4d, enrofloxacin last 15d, 4d, erythromycin last 15d, 4d, sulfadiazine last 15d, 4d, trimethoprim, neomycin last 20d, 5d, pefloxacin now, 7d |
| Chlortetracycline | 1 | > 1 MRL | F-G Broil | Farm | 0.77 | 24 | N | amoxicillin last 20d, 4d, [ciprofloxacin, pefloxacin] last 4d, 1d + now 3d, ciprofloxacin last 3d, 4d, amoxicillin now, 3d |
| Ciprofloxacin* | 1 | > 1 MRL | F-G Broil | Farm | 1.1 | 22 | N | amoxicillin last 19d, 3d, [ciprofloxacin, pefloxacin] now, 3d |
| Tilmicosin*, Ciprofloxacin* | 2 | > 1 MRL | F-G Broil | Farm | 2.6 | 36 | Y | amoxicillin last 15d, 2d, oxytetracycline last 3d, 3d, tilmicosin last 16d, 1d + last 20d, 1d + last 24d, 1d + last 30d, 1d, ciprofloxacin now, 1d, doxycycline now, 6d, tylosin now, 6d |
| Tilmicosin*, Ciprofloxacin* (enrofloxacin metabolite) | 2 | > 1 MRL | F-G Broil | Farm | 1.3 | 29 | N | amoxicillin last 7d, 3d + last 25d, 4d, enrofloxacin last 18d, 3d, tilmicosin last 7d, 3d |
| Sulfaquinoxaline | 1 | > 1 MRL | F-G Broil | Farm | 1.2 | 29 | N | ampicillin last 26d, 4d, enrofloxacin last 26d, 4d, levofloxacin last 12d, 3d |
| Sulfamethazine | 1 | > 1 MRL | F-G Broil | Farm | 1.9 | 29 | N | colistin last 8dm, 7d, enrofloxacin last 8d, 7d, neomycin now, 7d |
| Oxytetracycline* | 1 | > 1 MRL | F-G Broil | Farm | 1.9 | 32 | N | ciprofloxacin last 8d, 6d, oxytetracycline now, 7d |
| **Farms slow-growing broilers** |  |  |  |  |  |  |  |  |
| Oxytetracycline | 1 | > 1 MRL | Sonali | Farm | 1.0 | 60 | N | amoxicillin last 30d, 20d |
| Doxycycline*, Tilmicosin | 2 | > 1 MRL | Sonali | Farm | 0.72 | 50 | N | [tylosin, doxycycline] last 10d, 7, doxycycline now, 5d |
| Ciprofloxacin, Doxycycline* | 2 | > 10 MRL | Sonali | Farm | 0.70 | 60 | N | amoxicillin last 1d, 7d, colistin last 20d, 7d, doxycycline now, 5d |
| Tilmicosin | 1 | > 1 MRL | Sonali | Farm | 1.1 | 62 | Y | amoxicillin last 12d, 8d |
| Tilmicosin | 1 | > 1 MRL | Sonali | Farm | 0.9 | 65 | Y | none |
| Doxycycline**, Sulfadiazine** | 2 | > 1 MRL | Sonali | Farm | 0.84 | 66 | N | doxycycline last 15, 4d, [sulfadiazine, trimethoprim] last 30d, 4d, [erythromycin, sulfadiazine, trimethoprim] last 15d, 4d |
| Tilmicosin**, Oxytetracycline** | 2 | > 10 MRL | Sonali | Farm | 0.97 | 52 | N | amoxicillin last 45, 3d+ last 49d, 3d, doxycycline last 30d, 2d, neomycin last 13d, 1d, oxytetracycline last 30d, 3d, sulfaclozine last 13d, 3d, tilmicosin last 30d, 2d |
| Sulfadiazine*/trimethoprim*, Doxycycline* | 2 | > 10 MRL | Sonali | Farm | 0.59 | 61 | F | [amoxicillin, colistin] last 50d, 7d, amoxicillin last 15d, 4d, ciprofloxacin last 30d, 5d, doxycycline, now, 4d, [erythromycin, sulfadiazine, trimethoprim] now, 4d |
| Sulfaclozine | 1 | > 1 MRL | Sonali | Farm | 0.85 | 59 | F | amoxicillin last 35d, 5d, [erythromycin, sulfadiazine, trimethoprim] last 38d, 3d, neomycin last 39d, 4d, oxytetracycline last 65d, 3d + last 47d, 5d |
| Sulfaquinoxaline | 1 | > 1 MRL | Sonali | Farm | 1.524 | 51 | Y | amoxicillin last 10d, 9d, [erythromycin, neomycin, sulfadimidine, trimethoprim] now, 3d |
| Sulfaquinoxaline | 1 | > 1 MRL | Sonali | Farm | 1.025 | 52 | Y | amoxicillin last 16d, 7d, oxytetracycline last 5d, 7d |
|  |  |  |  |  |  |  |  |  |
| **Vietnam** | **Number of AM > 1 MRL** | **Level > MRL for highest concentration** | **Breed** | **Site type** | **Weight (kg)** | **Age** | **Finished**  **(Y/N)** | **Use** |
| **Markets** |  |  |  |  |  |  |  |  |
| Tilmicosin | 1 | > 1 MRL | Coloured | Market | 3 |  | Y | no treatment reported |
| Tilmicosin | 1 | > 1 MRL | F-G Broil | Market | 2.5 |  | Y | no treatment reported |
| Azithromycin (arbitrary threshold) | 1 | > 1 MRL | Coloured | Market | 2 |  | Y | no treatment reported |
| Oxytetracycline | 1 | > 1 MRL | Coloured | Market | 2.3 |  | Y | no treatment reported |
| Doxycycline | 1 | > 1 MRL | Coloured | Market | 2.9 |  | Y | no treatment reported |
| Doxycycline and Sulfaclozine | 2 | > 1 MRL | Coloured | Market | 2.3 |  | Y | no treatment reported |
| **Slaughterhouses** |  |  |  |  |  |  |  |  |
| Enrofloxacin and Ciprofloxacin | 1 | > 10 MRL | Coloured | Slaughterhouse | 2.6 |  | Y |  |
| **Farms slow-growing broilers** |  |  |  |  |  |  |  |  |
| Norfloxacin | 1 | > 1 MRL | Coloured | Farm | 2.2 | 120 | Y | [sulfonamide, trimethoprim] last 22, 5d, [doxycycline, florfenicol] last 30d, 5d, [ampicillin, colistin] last 45d, 3d |
| Florfenicol with metabolite | 1 | > 10 MRL | Coloured | Farm | 2.1 | 120 | Y | none |
| Sulfamonomethoxine* | 1 | > 1 MRL | Coloured | Farm | 1.2 | 70 | N | [sulfonamide, trimethoprim] last 5d, 7d, amoxicillin last 15d, 3d |
| Sulfamonomethoxine | 10 | > 10 MRL | Coloured | Farm | 1.4 | 70 | N | [doxycycline, florfenicol] last 30d, 3d |
| Sulfadimethoxine | 1 | > 1 MRL | Coloured | Farm | 3 | 120 | Y | none |
| Doxycycline | 1 | > 10 MRL | Coloured | Farm | 2.8 | 107 | N | [amoxicillin, colistin], previous unknown administration then, 3d |
| Doxycycline** | 1 | > 1 MRL | Coloured | Farm | 2.1 | 70 | N | sulfonamide last 45d, 4d, [doxycycline, florfenicol] last 25d, 3d, [amoxicillin, colistin] now, 4d |
|  |  |  |  |  |  |  |  |  |
| **Gujarat (India)** |  |  |  |  |  |  |  |  |
| **Markets** |  |  |  |  |  |  |  |  |
| Tilmicosin | 1 | > 1 MRL | F-G Broil | Market | 2.4 |  | Y | no treatment reported |
| Enrofloxacin | 1 | >10 MRL | Deshi | Market | 1.9 |  | Y | no treatment reported |
| Enrofloxacin | 1 | >10 MRL | Deshi | Market | 1.3 |  | Y | no treatment reported |
| Sulfadiazine and trimethoprim | 2 | > 1 MRL | F-G Broil | Market | 2.1 |  | Y | no treatment reported |
| **Farms fast-growing broilers** |  |  |  |  |  |  |  |  |
| Tilmicosin | 1 | > 1 MRL | F-G Broil | Farm | 1.7 | 36 | Y | enrofloxacin, last 27d, 3d |
| Chlortetracycline | 1 | > 1 MRL | F-G Broil | Farm | 2.4 | 40 | Y | enrofloxacin, last 30d, 3d |
| Sulfamethoxazole and trimethoprim | 2 | > 1 MRL | F-G Broil | Farm | 2.1 | 43 | Y | enrofloxacin, last 32d, 4d |
| Azithromycin (arbitrary threshold) | 1 | > 1 MRL | F-G Broil | Farm | 2.4 | 39 | Y | enrofloxacin, last 32d, 3d, oxytetracycline 35d, 3d |
| Chlortetracycline | 1 | > 1 MRL | F-G Broil | Farm | 2.3 | 35 | Y | enrofloxacin, last 32d, 2d |
| Sulfadiazine and trimethoprim | 2 | > 1 MRL | F-G Broil | Farm | 2.2 | 35 | Y | enrofloxacin, last 32d, 3d |
| Enrofloxacin | 1 | >10 MRL | F-G Broil | Farm | 1.6 | 37 | Y | enrofloxacin, last 34d, 3d |

Legend: N/A: not applicable, [drug 1, drug 2] last xd, yd: [therapeutic association], days since last use, duration in days. * non-compliant result that can be explained with log of AM use, ** non-compliant result for which there is evidence of administration of the specific AM but the time since last declared administration is too long to explain the residue result. If neither * or **: residue is not explained by treatment log or no log is available (endpoint). F-G Broil: fast-growing broiler, Coloured chicken in Vietnam and Sonali in Bangladesh: slow-growing broilers. Finished N: when, on farm, current age and weight were inferior to expected age and weight at sale.

**Supplementary Figure 1: Prevalence non-compliant chicken meat samples in Europe, data from Targeted sampling, group B1 (Antimicrobials).**

Prevalence of non-compliant chicken meat samples in Europe from non-suspicious samples (targeted sampling) were available for 14 years of data from the European Food Agency reports (EFSA): <https://food.ec.europa.eu/safety/chemical-safety/residues-veterinary-medicinal-products_en>. Prevalence in targeted sampling has been overall historically low (0·2%) and stabilising to 0·05%. The EU reports (2021-2022 data) concomitant to this study discussed in the manuscript appear as red bars. The annual number of samples tested within EU Member States varied between 15,000 and 19,000 samples; they were screening with a multianalyte method for antimicrobials detection (group B1).

**Supplementary Figure 2*:* Root cause analysis using a fishbone diagram pointing at illegitimate administration, feed or water contamination as causes of non-compliant results, i.e. at least one antimicrobial drug above maximum residue limit in a chicken meat sample.**


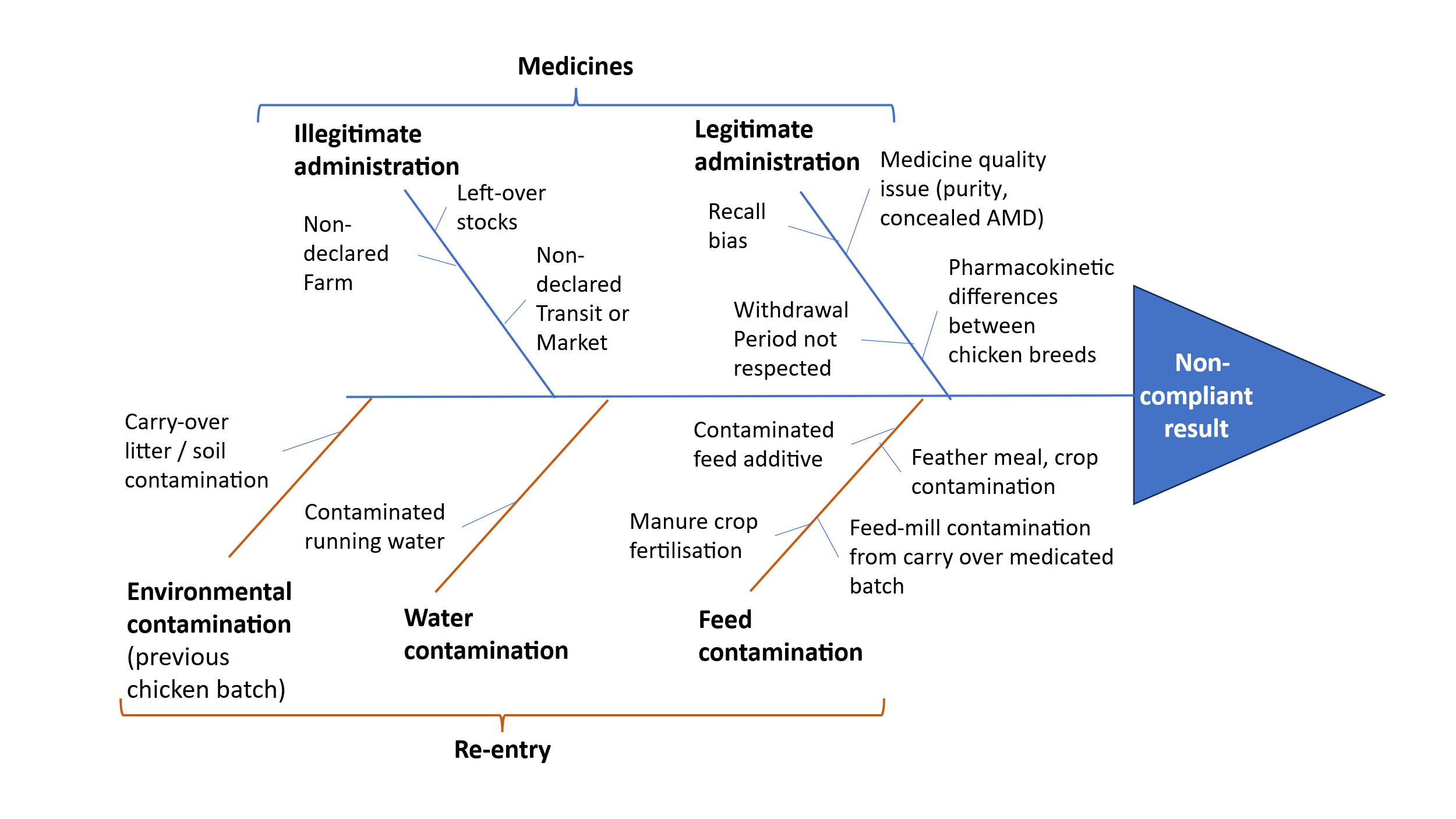


**Supplementary Figure 3.: Typical questionnaire applied on commercial farms (Gujarat example): sections relevant to antimicrobial use were selected herein**

1. **Farm composition**
   1. What is the maximum number of birds you can have on this farm? [integer] _________
   2. Do you practice all-in-all-out? [tick one box]

□ Yes □ No, multiple batches start and are harvested at different times

□ No, all batches starting at the same times, but fractions of the batches removed at different times

□ Other ____________________________________________________

- 1. Are chickens free-ranging? [one box]

□ Never, in shed/enclosure at all time □ For part of the day □ all day □ all day, all night

*If “Never”, “For part of the day”, “all day” to 3.1*

- - 1. How many poultry sheds/enclosures are there on the farm? [integer] ________
    2. How many sheds/enclosures are currently populated with poultry? [integer] _________
    3. What is the size of the area through which chickens free range? [text] __________________
    4. How far from farm buildings is the furthest free-ranging area? [text] _____ meters
    5. Are birds from other farms roaming on the same area? [one box]

|  | 3.4 which types of chicken, poultry species are currently on the farm | 3.5 How many are there on the farm **today**? | 3.6 Are there any in the same shed as the sampled batch? | 3.6 In the last 12 months, has there been ***other*** types of chickens, poultry species, on the farm? | 3.7 ***For each type selected in 3.4 and 3.6***, how many different batches did you raise in the last 12 months (24 months for laying hens)? |
| --- | --- | --- | --- | --- | --- |
| Exotic broiler chickens | □ |  |  | □ |  |
| Commercial Desi | □ |  | □ Yes □No | □ |  |
| Laying hens | □ |  | □ Yes □No | □ |  |
| Cockerel | □ |  | □ Yes □No | □ |  |
| Ducks (mallard-derived) | □ |  | □ Yes □No | □ |  |
| Muscovy ducks | □ |  | □ Yes □No | □ |  |
| Ducks (black desi backyard) | □ |  | □ Yes □No | □ |  |
| Ducks (White Desi backyard) | □ |  | □ Yes □No | □ |  |
| Geese | □ |  | □ Yes □No | □ |  |
| Quails | □ |  | □ Yes □No | □ |  |
| Other1 (Specify ________) | □ |  | □ Yes □No | □ |  |
| Other2 (Specify ________) | □ |  | □ Yes □No | □ |  |
| None |  |  |  | □ |  |

□ No, only this farm □ Yes, but not at the same time □ Yes, sometimes at the same time

- 1. What is the composition of the sampled batch?

| Breed | No. of chickens | Age when starting cycle | Current age (days) | Exp weight at sales (broiler) | No of eggs / year (layer) | Exp age at sales |
| --- | --- | --- | --- | --- | --- | --- |
| Exotic broiler |  |  |  |  |  |  |

1. **Feed, water**
   1. How do you feed your poultry? [multiple boxes]

□ Scavenging □ Household waste □ Ready commercial feed □ Loose feed (mixed by farmer)

- - 1. ***If ‘loose feed’ or ‘ready commercial feed’****,* What is the presentation of the feed?

□ Pellet □ Crumb □ Mash □ Other__________

- - 1. ***If ‘loose feed’****,* What is the composition of the feed? [text] _________________________________________________________________
    2. ***If ‘commercial feed’****,* Please show us the label of the feed bag/container, can we take a picture of the label? – **take a picture** and record the brand name if any [text] ____________________________________________________________
  1. Is any other product added to the feed **of the current batch**? [multiple boxes]

*If products have been added for the previous batches, specify in “Other”*

□ None □ Acidifiers □ Minerals □ Probiotics □ Sanitisers □ Vitamins

□ Antibiotics □ Don’t know □ Other________________

- - 1. ***If products are added to the feed****,* by whom? [multiple boxes]

□ By me □ By the feed dealer □ By feed company □ Other __________

- 1. Which source of water do you use for cleaning equipment, washing hands? [multiple boxes]

□ None □ Piped water (Municipality/Government) □ Piped water (private)

□ Well □ Pond □ River/lake □ Other ________

- 1. Which source of water do you use as drinking water for chickens? [multiple boxes]

□ Piped water (Municipality/Government) □ Piped water (private) □ Well

□ Pond □ River/lake □ Other ________

- 1. Do you add any other product in the drinking water? [multiple boxes]

□ None □ Acidifiers □ Minerals □ Probiotics □ Sanitisers □ Vitamins

□ Antibiotics □ Other_______________

- 1. Have you used, or planning to use, any vitamins, minerals, medicine, drugs or treatment on the **sampled** flock **TODAY**, including drugs which may already be mixed in the feed? [multiple boxes]
  2. Have you used **any other** products vitamins, minerals, medicine, drugs or treatment on the **sampled** flock **since the start of the production cycle**, including drugs which may already be mixed in the feed? [multiple boxes]

□ Yes, antibiobics □ Yes, vitamins □ Yes, minerals □ Yes, other drugs □ No

*6.2.1* ***If yes,* for antibiotics only**, please show us the packaging. Can we take pictures? Take a photo of each label/packaging. Fill the table below (if >5 antibiotics, use an additional table). **Ask also about the products for which packaging is not available**.

**Supplementary Figure 4*.:* Typical questionnaire applied at market level (Bangladesh example), sections relevant to antimicrobial use are presented herein:**

6.2 Do you use any vitamins, minerals, medicine, drugs, treatment on your chickens? [one box]

□ Yes □ No

6.2.1 ***If yes***, for which reasons? [text] _____________________________________

6.2.2 ***If yes***, If yes, please show us the packaging. Can we take pictures? [picture] – Take a photo of each label/packaging for **antibiotics only**, ignore others

6.2.3 ***If yes****,* having seen the packaging, is it an antibiotic?

□ Yes □ No □ Cannot tell/did not see the packaging

6.4 What do you do with sick birds?

□ Slaughter and sell as dressed meat □ Treat , with _____________ □ Dispose

□ Sell at lower price □ Eat □ Other________________

References for Supplementary Information

1 Hedges, S. *et al.* Antimicrobial residues in meat from chickens in Northeast Vietnam: analytical validation and pilot study for sampling optimisation. *Journal of Consumer Protection and Food Safety* **19**, 225–234 (2024). <https://doi.org/10.1007/s00003-024-01478-9>

2 Dubreil, E. *et al.* Validation approach for a fast and simple targeted screening method for 75 antibiotics in meat and aquaculture products using LC-MS/MS. *Food Addit Contam Part A Chem Anal Control Expo Risk Assess* **34**, 453–468 (2017). <https://doi.org/10.1080/19440049.2016.1230278>

3 Council of the European Union. *Council Directive 96/23/EC of 29 April 1996 on measures to monitor certain substances and residues thereof in live animals and animal products and repealing Directives 85/358/EEC and 86/469/EEC and Decisions 89/187/EEC and 91/664/EEC*, <<https://eur-lex.europa.eu/eli/dir/1996/23/oj>> (1996).

4 Commission Decision 2002/657. *Commission Decision of 12 August 2002 implementing Council Directive 96/23/EC concerning the performance of analytical methods and the interpretation of results*, <<https://eur-lex.europa.eu/eli/dec/2002/657/oj/eng>> (2002).

5 Community Reference Laboratories Residues. *Guidelines for the validation of screening methods (initial validation and transfer)*, 2010).

6 European Medical Agency. *Categorisation of antibiotics in the European Union*, <<https://www.ema.europa.eu/en/documents/report/categorisation-antibiotics-european-union-answer-request-european-commission-updating-scientific-advice-impact-public-health-and-animal-health-use-antibiotics-animals_en.pdf>> (2019).
